# ASSESSING MODERATED MEDIATION IN THE RELATIONSHIP BETWEEN SYMPTOM COUNTS, FALLS, AND HEALTH OUTCOMES

**DOI:** 10.1093/geroni/igad104.3182

**Published:** 2023-12-21

**Authors:** Michelle McKay, Margaret Brace

**Affiliations:** Villanova University, Villanova, Pennsylvania, United States; Villanova University, Villanova, Pennsylvania, United States

## Abstract

Almost half of older adults experience multiple symptoms that limit function and increase the risk for falls. The study aimed to determine if the relationship between symptoms (pain, endurance, weakness, balance, depression/anxiety) and outcomes (hospitalization, residential care, activity limitations, wellbeing, and mortality) are mediated by falls and whether symptoms further moderate outcomes of falls. Data from community-dwelling older adults (≥ 65 years, n=4921) enrolled in the National Health and Aging Trends Study (NHATS) 2015 cohort were used for analysis. Symptoms were categorized by symptom count (0, 1, 2, 3, 4, ≥5). Using generalized structural equation modeling, 2015 symptom counts were modeled to predict 2016 falls, 2016 health outcomes, and 2017 mortality. Falls partially mediated the association between symptoms in one year and outcomes the following year for all except residential care. In addition, the effect of falls on hospitalization is significantly moderated by symptoms (interaction of symptoms and fall OR=.89, robust SE=.045, p=.025). This dampening effect indicates that the overall effect of falls on hospitalization (OR=2.19, robust SE=.36, p<.001) is stronger at the lower levels of symptom count and weaker at the higher levels of symptom count. When there are ≥5 symptom present, there is no significant difference between the predicted probability of hospitalization for those who have not experienced a fall (.31, 95%CI: .28 to .34) and those who have fallen (.36, 95%CI: .31 to .41). Understanding the complex relationship between symptoms, falls, and outcomes can assist with developing interventions for symptom management and fall prevention.

